# Detection of *Pestivirus* in small ruminants in Central Java, Indonesia

**DOI:** 10.14202/vetworld.2021.996-1001

**Published:** 2021-04-24

**Authors:** W. Hidayat, H. Wuryastuty, R. Wasito

**Affiliations:** 1 Master Study Program, Faculty of Veterinary Medicine, Gadjah Mada University, Yogyakarta, Indonesia; 2 Department of Veterinary Internal Medicine, Faculty of Veterinary Medicine, Gadjah Mada University, Yogyakarta, Indonesia; 3 Department of Veterinary Pathology, Faculty of Veterinary Medicine, Gadjah Mada University, Yogyakarta, Indonesia

**Keywords:** antibody enzyme-linked immunosorbent assay, bovine viral diarrhea virus, pestivirus, reverse transcriptase-polymerase chain reaction, small ruminants

## Abstract

**Background and Aim:**

Globally, pestiviruses are among the most economically important viral pathogens of livestock. The genus *Pestivirus* comprises four species, including bovine viral diarrhea virus type 1 and 2 (BVDV-1 and BVDV-2), which infect cattle, border disease virus and classical swine fever virus which infect small ruminants and pigs, respectively. Accumulating evidence suggests that pestiviruses are no longer species-specific, creating new challenges for disease control. In Indonesia, investigations related to pestiviruses remain focused on cattle as the primary host and no research has been conducted on small ruminants (sheep and goats). Therefore, the present study aimed to study the possible occurrence of pestivirus (BVDV or BVD) infections in small ruminants in Indonesia, particularly in Central Java.

**Materials and Methods:**

We used 46 blood samples consisting of 26 sheep’s blood and 20 goat’s blood. Samples were selected from 247 small ruminant blood collected between July and October 2020 in Central Java, Indonesia, which met the following criteria: Female, local species, approximately 1-2 years old, never been pregnant, raised in the backyard, and had no close contact with cattle in either shelter or grazing area. We tested plasma samples from sheep and goats using competitive antibody enzyme-linked immunosorbent assay to detect specific antibodies against pestivirus followed by reverse transcription-polymerase chain reaction (RT-PCR) analysis for all positive samples to differentiate the species of pestivirus.

**Results:**

Two of the 20 samples collected from goats were positive for pestivirus at the serological and molecular levels, whereas 2 of 26 samples collected from sheep were doubtful but tested negative by RT-PCR. The genotyping test results obtained using nested PCR revealed that the positive samples collected from goats had a BVDV-1 genotype.

**Conclusion:**

The results of the present study demonstrated that BVDV-1 can infect species other than bovines, in Central Java, Indonesia. Further studies involving a larger number of samples are required to: (1) Determine the actual seroprevalence of pestiviruses in small ruminants and (2) Determine the potency of small ruminants as reservoirs for pestiviruses, both of which are important for the identification of the appropriate control program for pestiviruses in Indonesia.

## Introduction

According to the International Committee on Taxonomy of Viruses [1], the genus *Pestivirus* family Flaviviridae comprises four species, including bovine viral diarrhea virus types 1 and 2 (BVDV-1 and BVDV-2), which infect cattle, border disease virus (BDV), and classical swine fever virus which infect small ruminants and pigs, respectively. Because of their impact on milk production and reproductive efficiency as well as on the general health status of the animals in the herd, all pestiviruses can cause serious economic losses in the livestock industry [2]. Genetically, pestiviruses are positive-sense single-stranded ribonucleic acid (RNA) viruses with genomes that are approximately 12.5 kb in length and are organized as an open reading frame encoding a single polyprotein composed of 4000 amino acids and flanked on both sides by 5’-and 3’-non-coding regions. The four pestivirus species are genomically and antigenically very similar to each other [3].

Historically, the taxonomic classification of pestiviruses was based on the host species they were isolated from, where any pestivirus infection affecting sheep and goats was considered as BDV without supportive testing. Therefore, in older studies, it is unclear whether the isolated pestivirus was BVDV or BDV [4,5]. At present, it has been reported that pestiviruses are not strictly host-specific [6]. Serologically, there is increasing evidence that BVDV-1 infection occurs in more than 40 other species of both domestic and wild artiodactyl, such as giraffe, deer, impala, African buffalo, as well as goats and sheep, and produces similar clinical symptoms [7-9]. Among these species, domestic small ruminants, consisting of both goats and sheep, are a major concern of experts because of their potential as carriers and transmitters of BVDV. This is because sheep and goats come into contact with cows more often than other species, thereby facilitating disease transmission between species [10-13]. Heterogeneity in the host species can seriously affect the livestock industry and provide new challenges, especially in diagnosis and disease control [6].

In Indonesia, BVD in cattle was first diagnosed in 1989 [14]. The prevalence of the disease continues to increase over the years. The seropositivity of BVDV-1 among beef, dairy, and breeding cattle populations varies from approximately 46-56.2% [15,16]. Because BVDV-1 from cattle is the only officially recognized pestivirus species in Indonesia, all studies related to this virus are focused on cattle as the natural host and no research on pestiviruses in small ruminants has been conducted.

The present study aimed to serologically and molecularly study the possible occurrence of a pestivirus infection in small ruminants in Indonesia, particularly in Central Java.

## Materials and Methods

### Ethical approval

The present study protocol was approved by the Ethical Research Committee, Faculty of Veterinary Medicine, Gadjah Mada University, Yogyakarta, Indonesia, No.: 0074/EC-FKH/Int./2020.

### Study period and location

Samples were collected from July to October 2020 from Central Java, Indonesia. Samples were processed at Veterinary Internal Medicine Laboratory, Faculty of Veterinary Medicine, Gadjah Mada University.

### Samples

We selected 247 small ruminants (sheep and goat) using the cluster random sampling method. which met the following criteria: Female, local species, approximately 1-2 years old, never been pregnant, raised in the backyard, and had no close contact with cattle in either shelter or grazing area. We used 46 blood samples consisting of 26 from sheep and 20 from goats. Three milliliters of whole blood were withdrawn from the jugular vein using ethylenediaminetetraacetic acid (K_3_EDTA)-BD Vacutainer^®^ tubes (Becton Dickinson and Company, NJ, USA). The plasma obtained after centrifuging the blood samples at 1500× *g* for 15 min was stored at −20°C until the assay was conducted.

### Competitive antibody enzyme-linked immunosorbent assay

We individually assayed the plasma samples for the presence of specific antibodies against protein NS2-3 (p80) using a commercially available competitive Ab-ELISA kit (IDEXX BVDV p80 Protein Antibody Test Kit) (IDEXX Laboratories, Inc., Westbrook, ME, USA). This test allows for the detection of anti-BVDV, mucosal disease, and anti-BDV antibodies in ruminants and is based on the competition between pestivirus antibodies present in the sample and a peroxidase coupled monoclonal anti-p80-antibody (WB112). To detect the anti-BVD antibodies, positive and negative controls were first diluted 1:1 while all of the plasma samples were directly diluted 1:4 using dilution buffer in the appropriate wells and then incubated for 24 h at 4°C. Subsequently, the wells were washed 3-5 times using 300 mL of washed buffer, after which 100 mL of conjugated anti-bovine HRP was added to each well and incubated for 30 min at room temperature (25ºC). Then, the plates were washed as previously described to remove the excess conjugate. For color development, 100 mL of 3,3’,5,5’-tetramethylbenzidine substrate was added into each well, and then, the samples were incubated in darkness for 10 min. The reaction was terminated by adding 100 mL of stop solution to each well, after which the absorbance at 450 nm was measured using an ELISA reader. The ELISA results were expressed as sample-to-negative ratios, which were calculated as described in the manufacturer’s instructions [15]. The test is valid when the percentage of inhibition of the negative and positive controls is ≥80% and <20%, respectively. Samples showing inhibition of ≤40%; 40%–50%, and ≥50% were considered positive, doubtful, and negative, respectively. All positive samples were assessed by polymerase chain reaction (PCR) analysis to differentiate the species of pestivirus.

### RNA extraction and one-step RT-amplification

Pestivirus RNA was extracted using a commercially available kit (Viral Nucleic Acid Extraction Kit II, Geneaid Biotech Ltd., Taiwan) following the manufacturer’s instructions. The extracted RNA was subjected to reverse transcription and PCR amplification in one-step reactions using the MyTaq™ One-Step RT-PCR kit (Bioline Meridian Bioscience, Australia) according to the manufacturer’s instructions with a Biometra Personal Combi thermocycler (37079 Gottingen, Germany). Four different PCR assays were performed to detect general panpestivirus [17], BDV [18], BVDV-1, and BVDV-2 [19] amplifying the corresponding gene targets. The primer sequences used to detect general panpestivirus, BDV, BVDV-1, and BVDV-2 in the present study are presented in Table-1 [17-19]. BVDV-1 strain Singer was used as a positive control while sterile distilled water was used in place of template DNA for the negative control.

**Table 1:** Oligonucleotide primers used in the present study.

Primers	Target species	Sequence (5’–3’)	Amplicon length (bp)	References
324	BVDV	5’ ATG CCC WTA GTA GGA CTA GCA 3’	288	[17]
326		5’ TCA ACT CCA TGT GCC ATG TAC 3’		
PBD1	BDV	5’ TCG TGG TGA GAT CCC TGA G 3’	225	[18]
PBD2		5’ GCA GAG ATT TTT TAT ACT AGC CTA TRC 3’		
Set A forward	BVDV-1	5’ GTA GTC GTC AGT GGT TCG 3’	198	[19]
Set A reverse		5’ GCC ATG TAC AGC AGA GAT 3’		
RB21	BVDV-2	5’ CGA CAC TCC ATT AGT TGA GC 3’	105	[19]
RB22		5’ GTC CAT AAC GCC ACG AAT AG 3’		

Using panpestivirus generic primers (324/326) and BDV-specific primers (PBD1/PBD2) for the corresponding 5’ untranslated region (5’ UTR), the expected sizes of the PCR amplification products were 288 and 225 bp, respectively. The thermal cycling conditions were as follows: 30 min of reverse transcription at 50°C followed by 5 min of initial denaturation at 95°C and 35 cycles of denaturation at 94°C for 30 s, annealing at 50°C for 30 s and elongation at 72°C for 45 s, and then final elongation at 72°C for 5 min.

BVDV genotyping was performed using nested PCR. The first step of reverse transcriptase PCR amplification was performed using a MyTaq™ One-Step RT-PCR kit (Bioline Meridian Bioscience, Australia) following the manufacturer’s instructions. The thermal cycling conditions for the first amplification were as follows: Reverse transcription at 42°C for 1 h, followed by an initial denaturation at 94°C for 3 min and by 30 cycles of denaturation at 94°C for 30 s, annealing at 50°C for 45 s, and elongation at 72°C for 1 min, with a final elongation at 72°C for 10 min. The DNA products of the first amplification were then used as templates for the second round of amplification. The PCR mixture contained all the component used in the first amplification but without RT enzyme and the primers used to amplify BVDV-1 were substituted with a specific primer pair for BVDV-2. The cycle conditions for the second amplification were as follows: An initial denaturation at 94°C for 2 min followed by 30 cycles of denaturation at 94°C for 30 s, annealing at 50°C for 45 s, and elongation at 72°C for 1 min with a final elongation step at 72°C for 7 min.

Following PCR amplification, the products were separated by 1.5% agarose gel electrophoresis at 120 V 400 A for 45 min. The agarose gel was then immersed in a 1× buffer Tris-borate-EDTA™ buffer (Sigma-Aldrich) supplemented added with 0.5 mg/mL of ethidium bromide™ (Sigma-Aldrich) solution for 15 min. The stained electrophoresed PCR product was then visualized under an ultraviolet transilluminator and imaged using the Gel Logic 100 Imaging System (Kodak, Carestream Health, Inc. Rochester, NY 14608).

## Results

In the present study, all sampled animals were locally raised and belonged to different smallholder farmers. The animals did not also share the same grazing area or shelter with cattle. Some of the animals examined in the present study exhibited an unthrifty condition with an average body condition score of 2-5. However, none of the animals (n=46) exhibited any specific clinical symptoms of BVDV infection. Using a competitive antibody enzyme-linked immunosorbent assay (Ab-ELISA), of 20 samples collected from goats, 2 tested positive and 18 tested negative with observed percentages of inhibition of 28.68% and 32.73%, respectively, whereas, of the 26 samples collected from sheep, two were doubtful but tested negative by RT-PCR. The Ab-ELISA used in the present study can detect antibodies for both BVDV and BDV which can infect both cattle and small ruminants (sheep and goats). Thus, the test cannot provide results for specific types of antibodies that the tested animals produced.

**Figure-1:**
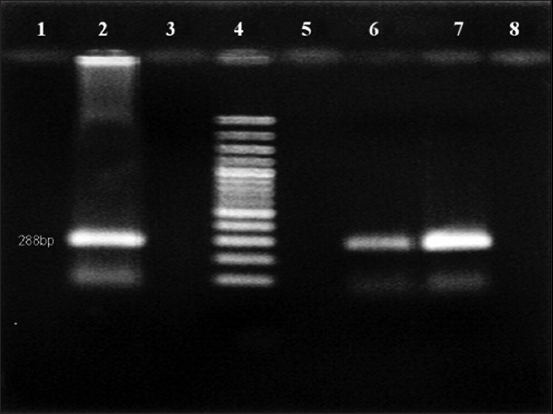
Reverse transcription-polymerase chain reaction analysis of samples for general pestiviruses. Lane 2: Positive control, lane 3: Negative control, lane 4: 100 bp DNA marker, lanes 6-7: Pestivirus positive specimen.

**Figure-2:**
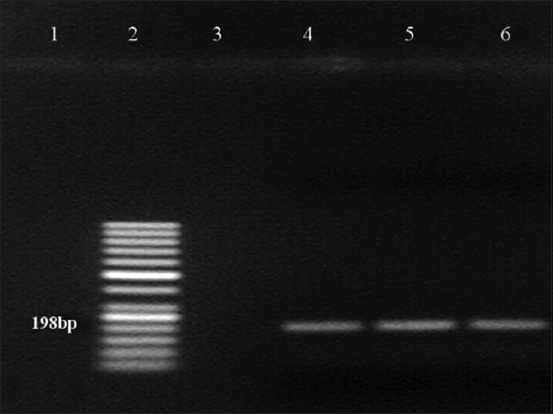
Reverse transcription-polymerase chain reaction test of samples for bovine viral diarrhea virus type 1 (BVDV-1). Lane 2: 50 bp DNA marker, lane 3: negative control, lane 4: Positive control, lanes 5-6: BVDV-1-positive specimen.

**Figure-3:**
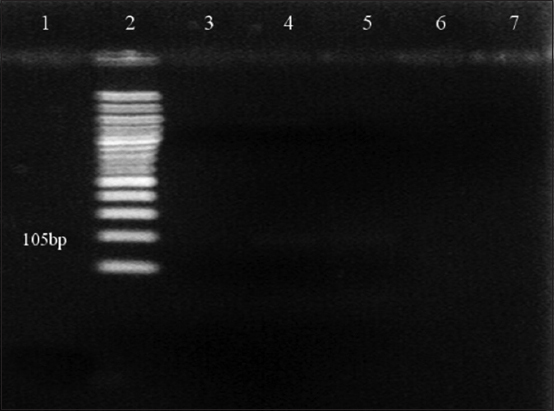
Agarose gel electrophoresis (1.5%) of polymerase chain reaction (PCR) amplified products using species-specific PCR primer sets for bovine viral diarrhea virus type 2 (BVDV-2) amplification. Lane 2: 100 bp DNA marker. PCR product of BVDV-2 was not detected in any of the samples, including the positive control.

**Figure-4:**
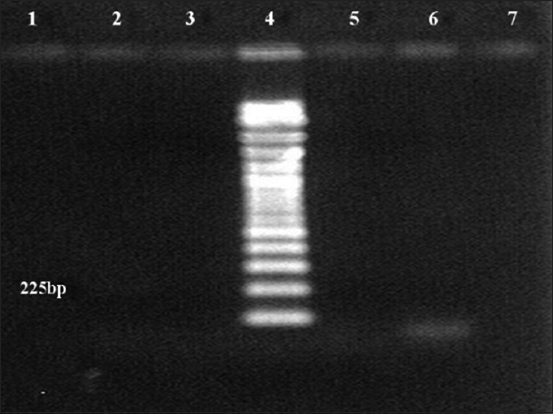
Agarose gel electrophoresis (1.5%) of polymerase chain reaction (PCR) amplified products using species-specific PCR primer sets for border disease virus (BDV) amplification. Lane 4: 100 bp DNA marker. No PCR product for BDV was detected in any samples, including the positive control.

As shown in Figure-1, amplification of 5’UTR using the general pestivirus primers 324 and 326 resulted in the successful detection of viral RNA from the blood samples of two goats at 288 bp. The genotyping test results using nested PCR revealed that the positive samples collected from goats had BVDV-1 genotype (Figure-2). However, no PCR product was detected with other pestiviruses (Figures-3 and 4).

## Discussion

In the present study, we evaluated the presence of *Pestivirus* infection in small ruminants by collecting blood samples from unvaccinated sheep and goats using the criteria explained in the Materials and Methods section and serologically assessed these with a competitive Ab-ELISA. ELISA is a reliable technique for the detection of antibodies against the highly conserved non-structural BVDV protein NS2-3, a protein that is produced in large quantities during viral replication after BVDV infection. In unvaccinated animal flocks, serological testing is a convenient technique for disease prevalence screening since the antibodies detected represent natural infections and are not due to vaccination. It was previously shown that a single animal testing positive by Ab-ELISA is sufficient to reveal the presence of animals with specific antibodies against a disease [15,20,21]. The sheep and goats sampled in the present study were approximately >1 year of age, demonstrating that antibodies were generated as a result of natural exposure from the environment, since the animals were no longer under the influence of maternal antibodies. The prevalence rates of pestiviruses in small ruminants in large flocks vary from 20% to 65% depending on the location within the country and the type of husbandry [22,23]. The prevalence of *Pestivirus* antibodies in goats is generally lower and has been reported to range from 10% to 25% [24]. According to the results of a previous study [11], the low prevalence of *Pestivirus* antibodies could be due to the slow natural spread of pestiviruses within and between goat herds, since goats do not appear to be an efficient host for ruminant pestiviruses. In the present study, 2 of 26 sheep were considered to yield doubtful results in the Ab-ELISA but tested negative by RT-PCR. According to the result of one previous study [9], interpretation of the results is largely dependent on the negative control value when utilizing an Ab-ELISA. The percentage of inhibition of the sample is the ratio between the sample absorbance value and the average of two negative control absorbance values multiplied by 100. Therefore, the lower the average negative control value is, the higher the percentage of sample inhibition leading to false-negative results. The manufacturer provided the validity criteria used in the present study.

Of the 20 goats tested, 2 (10%) tested positive at the serological and molecular levels. This result indicates that the pestiviruses have already been present in a small ruminant population, in Central Java, Indonesia. To date, there has been no published report related to the presence of BVDV infection in small ruminants in Indonesia (sheep and goats). The low prevalence of antibodies in the present study could be related to the limited samples tested and, therefore, may not be representative of the small ruminant population in Indonesia. However, these initial findings should not be ignored, since: (1) Indonesia has the largest small ruminant population in Southeast Asia and (2) pestiviruses are not strictly species-specific and can, therefore, be transmitted to a variety of even-toed ungulate [25,26]. All of these factors could create serious economic impacts in livestock industries in the near future.

The PCR results obtained for the positive samples using the general pestivirus primers 324 and 326, which bind to the 5’UTR of the virus causing BVD, are presented in Figure-1. Two positive samples indicate the presence of an amplification product at the 288 bp position-specific for BVDV. However, the amplification results of sheep plasma using the same pair of primers were all negative. The genotyping test results using nested PCR revealed that the positive samples collected from goats had BVDV-1 genotype (Figure-2) but there was no evidence for the presence of BVDV-2 (Figure-3) and BDV (Figure-4). This result is in agreement with the finding obtained in a previous study [27] where BVDV-1 was reported to be a causative agent for pestivirus infection of diseased goats in Southwest China. However, the blood samples tested in the present study were collected from clinically healthy small ruminants.

In infected animals, the clinical manifestations caused by BVDV are primarily related to reproductive inefficiency and reduction in milk yield resulting in significant economic losses to the cattle industry worldwide [28]. However, the most devastating consequence of BVDV infection is the presence of cows with persistent BVDV infection. Cows with IP-BVDV are immunotolerant and continuously shed the virus and transmit it rapidly and continuously throughout their life through direct contact with other sensitive and unvaccinated animals. In acutely infected animals, the duration of pestivirus infection is typically short and few viruses are only intermittently shed. Some studies have demonstrated that transient infections are frequently asymptomatic or are sometimes accompanied by mild respiratory or enteric symptoms, demonstrating that acutely infected animals inefficiently transmit the virus to susceptible animals. However, based on the result of a previous study [11], goats may still be a source of infection especially when they are maintained in close contact. In practice, a herd is not considered infected with BVDV until persistently infected animals are discovered [10].

At the molecular level, BVDV has been differentiated into two genotypes: BVDV-1 and BVDV-2 [29]. Each genotype can be further divided into two biotypes, cytopathic and non-cytopathic based on their cytopathogenicity on cell culture [30]. There are many reports on genetic variations of BVDV from many countries [31,32]. However, BVDV-1 remains the dominant genotype that infects cattle and is spreading worldwide [23,33]. The results of the present study provide further evidence that BVDV-1 infection has occurred in species other than bovines. BVDV-1 is now redesignated as *Pestivirus* A [34].

The origin of the virus observed in the present study is not known since the *Pestivirus* infection detected in two goats occurred without any contact with either cattle or sheep, indicating the circulation of the virus among them without contact with cattle or sheep. Given that these viruses are clinically difficult to recognize, it is possible that the virus existed in the goats without being noticed. Based on the results of a previous study [25], the primary source of virus is typically unknown for BVDV infection in species other than bovines. In the epidemiology of *Pestivirus* infections, factors such as the presence of PI animals, uncontrolled animal movement, interspecies transmission, sharing grazing areas, and water sources during pasturing or the purchase of new animals to renew breeding stock have been considered important sources for the introduction of pathogens into farms or flocks [8,35,36]. Similar results were reported in a previous study [24] where BVDV was suggested to have the potential to persist in the goat population.

## Conclusion

The results of the present study provide evidence that BVDV-1 infection has occurred in species other than bovines, in Central Java, Indonesia. However, additional studies involving a larger number of samples are required to: (1) Determine the real seroprevalence of pestiviruses in small ruminants and (2) reveal the potency of small ruminants as reservoirs for pestiviruses, both of which are important for the identification of the appropriate control program for pestiviruses in Indonesia.

## Authors’ Contributions

WH contributed to blood sampling and ELISA. HW performed the RNA extraction and RT-PCR analysis. HW and RW supervised the study. All authors read and approved the final manuscript.

## References

[1] International Committee on Taxonomy of Viruses (2016). Virus Taxonomy:2015.

[2] Walz P (2015). Diseases of the Alimentary Tract. I. Bradford Smith (Red.). Large Animal Internal Medicine.

[3] Tautz N, Tews B.A, Meyers G (2015). The molecular biology of pestiviruses. Adv. Virus Res.

[4] Braun U, Hilbe M, Peterhans E, Schweizer M (2019). Border disease in cattle. Vet. J.

[5] Giangaspero M, Ibata G, Savini G, Osawa T, Tatami S, Takagi E, Moriya H, Okura N, Kimura A, Harasawa R (2011). Epidemiological survey of border disease virus among sheep from northern districts of Japan. J. Vet. Med. Sci.

[6] Passler T, Walz P.H (2010). Bovine viral diarrhea virus infections in heterologous species. Anim. Health Res. Rev.

[7] Broaddus C.C, Lamm C.G, Kapil S, Dawson L, Holyoak G.R (2009). Bovine viral diarrhea virus abortion in goats housed with persistently infected cattle. Vet. Pathol.

[8] Albayrak H, Gumusova S.O, Ozan E, Yazici Z (2012). Molecular detection of pestiviruses in aborted foetuses from provinces in Northern Turkey. Trop. Anim. Health Prod.

[9] Czopowicz M, Kaba J, Schirrmejer H, Bagnicka E, Szalus-Jordanow O, Nowicki M, Witkowski L, Frymus T (2011). Serological evidence for BVDV-1 infection in goats in Poland-short communication. Acta Vet. Hung.

[10] Han Y.J, Chae J.B, Chae J.S, Yu D.H, Park J, Park B.K, Kim H.C, Yoo J.G, Choi K.S (2016). Identification of bovine viral diarrhea virus infection in Saanen goats in the Republic of Korea. Trop. Anim. Health. Prod.

[11] Lysholm S (2017). Prevalence and Risk Factors for BVDV in Goats and Cattle in and Around Gaborone, Botswana.

[12] Lysholm S, Ramabu S.S, Berg M, Wensman J.J (2019). First-time detection of bovine viral diarrhea virus, BVDV-1, in cattle in Botswana. Onderstepoort J. Vet. Res.

[13] Mishra N, Rajukumar K, Tiwari A, Nema R.K, Behera S.P, Satav J.S, Dubey S.C (2011). Prevalence of bovine viral diarrhea virus (BVDV) antibodies among sheep and goats in India. Trop. Anim. Health Prod.

[14] Wiyono A, Ronohardjo P, Graydon R, Daniels P (1989). Diare ganas sapi:Kejadian penyakit pada sapi Bali bibit asal Sulawesi. Penyakit Hewan [Mucosal disease in cattle:1. Disease incidence in Balinese cattle from South Sulawesi that just arrived in West Kalimantan. Animal Diseases.

[15] Wuryastuti H, Putro P.P, Wasito R (2016). Prevalence of antibody to bovine viral diarrhea field virus in dairy cattle in Central Java, Indonesia. In:Proceeding of 19^th^ Federation of Asian Veterinary Association Congress, Ho Chi Minh City, Vietnam.

[16] Irianingsih S.H, Wuryastuty H, Wasito R, Wibawa H, Tjatur Rasa F.S, Poermadjaja B (2019). Genetic analysis of NS5B gene from bovine viral diarrhea virus-infected cattle in Central and East Java, Indonesia. Vet. World.

[17] Vilcek S, Nettleton P.P, Paton D.J, Belak S (1997). Molecular characterization of ovine pestiviruses. J. Gen. Virol.

[18] Vilcek S, Paton D.J (2000). A RT-PCR assay for the rapid recognition of border disease virus. Vet. Res.

[19] Ridpath J.F, Bolin S.R, Dubovi E.J (1994). Segregation of bovine viral diarrhea virus into genotypes. Virology.

[20] Feknous N, Hanon J.B, Tignon M, Khaled H, Bouyoucef A, Cay B (2018). Seroprevalence of border disease virus and other pestiviruses in sheep in Algeria and associated risk factors. BMC Vet. Res.

[21] Tao J, Liao J, Wang Y, Zhang X, Wang J, Zhu G (2013). Bovine viral diarrhea virus (BVDV) infections in pigs. Vet. Microbiol.

[22] De Mia G.M, Greiser-Wilke I, Feliziani F, Giammarioli M, De Giuseppe A (2005). Genetic characterization of a caprine pestivirus as the first member of a putative novel pestivirus subgroup. J. Vet. Med. B Infect. Dis. Vet. Public Health.

[23] Kaiser V, Nebel L, Schupbach-Regula G, Zanoni R.G, Schweizer M (2017). Influence of border disease virus (BDV) on serological surveillance within the bovine virus diarrhea (BVD) eradication program in Switzerland. BMC Vet. Res.

[24] Bachofen C, Vogt H.R, Stalder H, Mathys T, Zanoni R, Hilbe M, Schweizer M, Peterhans E (2013). Persistent infections after natural transmission of bovine viral diarrhea virus from cattle to goats and among goats. Vet. Res.

[25] Udo H.M.J, Budisatria I.G.S (2011). Fat-tailed sheep in Indonesia;an essential resource for smallholders. Trop. Anim. Health Prod.

[26] Nelson D.D, Duprau J.L, Wolff P.L, Evermann J.F (2016). Persistent bovine viral diarrhea virus infection in domestic and wild small ruminants and camelids including the mountain goat (*Oreamnos americanus*). Front. Microbiol.

[27] Deng Y, Wang S, Liu R, Hao G (2018). Genetic diversity of bovine viral diarrhea virus infection in goats in Southwestern China. J. Vet. Med.

[28] Heauer C, Healy A, Zerbini C (2007). Economic effects of exposure to bovine viral diarrhea virus on dairy herds in New Zealand. J. Dairy Sci.

[29] Ridpath J.F (2005). Practical significance of heterogeneity among BVDV strains:Impact of biotype and genotype on U. S. control programs. Prev. Vet. Med.

[30] Ammari M, McCarthy F.M, Nanduri B, Heazlewood J (2012). Understanding the pathogenesis of cytopathic and noncytopathic bovine viral diarrhea virus infection using proteomics. Proteomics Application in Biology.

[31] Zhang J.J, Ma C, Li X.Z, Liu Y.X, Huang K, Cao J (2013). Genetic diversity of bovine viral diarrhoea virus in Beijing region, China from 2009 to 2010. Afr. J. Microbiol. Res.

[32] Bazzucchi M, Bertolotti L, Giammaroli M, Rossi E, Petrini S, Rosati S, De Mia G.M (2017). Complete genome sequence of a bovine viral diarrhea vieus sub-genotype 1g strain isolated in Italy. Viruses.

[33] Yesilbag K, Alpay G, Becher P (2017). Variability and global distribution of sub-genotypes of bovine viral diarrhea virus. Viruses.

[34] Ricci S, Bartolini S, Morandi F, Cuteri V, Preziuso S (2019). Genotyping of Pestivirus A (bovine viral diarrhea virus 1) detected in faeces and in other specimens of domestic and wild ruminants at the wildlife-livestock interface. Vet. Microbiol.

[35] Sevik M (2018). The role of pestivirus (BDV and BVDV) in ruminant abortion cases in the Afyonkarahisar province. Kocatepe Vet. J.

[36] Qi L, Beaunee G, Amoux S, Dutta B.L, Joly A, Vergu E, Ezanno P (2019). Neighborhood contacts and trade movements drive the regional spread of bovine viral diarrhea virus (BVDV). Vet. Res.

